# Pediatric Therapeutic Drug Monitoring for Selective Serotonin Reuptake Inhibitors

**DOI:** 10.3389/fphar.2021.749692

**Published:** 2021-10-01

**Authors:** Jeffrey R. Strawn, Ethan A. Poweleit, Chakradhara Rao S. Uppugunduri, Laura B. Ramsey

**Affiliations:** ^1^ Anxiety Disorders Research Program, Department of Psychiatry and Behavioral Neuroscience, University of Cincinnati, Cincinnati, OH, United States; ^2^ Division of Clinical Pharmacology, Cincinnati Children’s Hospital Medical Center, Department of Pediatrics, University of Cincinnati College of Medicine, Cincinnati, OH, United States; ^3^ Division of Child and Adolescent Psychiatry, Department of Pediatrics, Cincinnati Children’s Hospital Medical Center, Cincinnati, OH, United States; ^4^ Division of Research in Patient Services, Cincinnati Children’s Hospital Medical Center, Department of Pediatrics, University of Cincinnati College of Medicine, Cincinnati, OH, United States; ^5^ Division of Biomedical Informatics, Cincinnati Children’s Hospital Medical Center, Cincinnati, OH, United States; ^6^ Department of Biomedical Informatics, University of Cincinnati College of Medicine, Cincinnati, OH, United States; ^7^ CANSEARCH Research Platform in Pediatric Oncology and Hematology, Department of Pediatrics, Gynecology and Obstetrics, University of Geneva, Geneva, Switzerland

**Keywords:** therapeutic drug monitoring, selective serotonin reuptake inhibitor, depressive disorder, anxiety disorder, tolerability, pediatric, child and adolescent psychiatry

## Abstract

Therapeutic drug monitoring (TDM) is uncommon in child and adolescent psychiatry, particularly for selective serotonin reuptake inhibitors (SSRIs)—the first-line pharmacologic treatments for depressive and anxiety disorders. However, TDM in children and adolescents offers the opportunity to leverage individual variability of antidepressant pharmacokinetics to shed light on non-response and partial response, understand drug-drug interactions, evaluate adherence, and characterize the impact of genetic and developmental variation in pharmacokinetic genes. This perspective aims to educate clinicians about TDM principles and examines evolving uses of TDM in SSRI-treated youths and their early applications in clinical practice, as well as barriers to TDM in pediatric patients. First, the impact of pharmacokinetic genes on SSRI pharmacokinetics in youths could be used to predict tolerability and response for some SSRIs (*e.g.*, escitalopram). Second, plasma concentrations are significantly influenced by adherence, which may relate to decreased efficacy. Third, pharmacometric analyses reveal interactions with proton pump inhibitors, oral contraceptives, cannabinoids, and SSRIs in youths. Rapid developments in TDM and associated modeling have enhanced the understanding of variation in SSRI pharmacokinetics, although the treatment of anxiety and depressive disorders with SSRIs in youths often remains a trial-and-error process.

## Introduction

Therapeutic drug monitoring (TDM)—the determination of medication concentrations in patients with the goal of optimizing medication dosing—is uncommon in child and adolescent psychiatry, potentially owing to numerous barriers that have limited its adoption into clinical practice.

Selective serotonin reuptake inhibitors (SSRIs) are the mainstay of pharmacologic treatment for pediatric depressive (Goodyer and Wilkinson, 2019) and anxiety disorders (Strawn et al., 2020b), as well as obsessive compulsive disorder (OCD) (Watson and Rees, 2008). SSRI dosing in children and adolescents generally relies on a ‘one size fits all’ approach. Clinicians often initiate antidepressants at low-doses and slowly titrate these medications until either encountering a side effect or response. If intolerable side effects occur, the SSRI dose is decreased, or the medication is discontinued. Moreover, the initial SSRI dose is often based on the dosages used in clinical trials and the clinician’s comfort with titration. The dose a clinician targets for an individual patient is frequently the mean dose used in clinical trials and the adequacy of antidepressant treatment trials for individual patients is based on target doses (Brent et al., 2008; Strawn et al., 2020a), which fail to account for adherence and variation in drug exposure.

In contemporary clinical practice, factors that influence antidepressant exposure have not yet been incorporated into treatment guidelines for pediatric anxiety (Walter et al., 2020) and depressive disorders (Cheung et al., 2007). Moreover, many psychiatric clinicians contend that circulating antidepressant concentrations are unrelated to response (Ruhé et al., 2006). However, this conflicts with recommendations to titrate SSRI dose in patients with partial responses (Dwyer et al., 2020) and to consider lowering doses in patients with tolerability concerns (Wilens et al., 2003; Luft et al., 2018). Further, intrinsic factors that affect drug concentrations are rarely considered in clinical trials of antidepressants in youth.

Given the current approach to dosing SSRIs and increasing evidence linking variation in SSRI exposure and differences in efficacy and tolerability, TDM may have increasing utility in child and adolescent psychiatry. TDM offers the opportunity to leverage individual variability of antidepressant pharmacokinetics to: 1) shed light on non-response and partial response (Sakolsky et al., 2011); 2) understand drug-drug interactions (Vaughn et al., 2021); 3) evaluate adherence (Fekete et al., 2020); and 4) understand the impact of genetic and developmental variation in pharmacokinetic genes (Strawn et al., 2020c). With these considerations in mind, this Perspective introduces clinicians to TDM principles and illustrates TDM applications in child and adolescent psychiatry. In parallel, this Perspective introduces pharmacologists to the complexity of exposure-response and exposure-tolerability relationships in child and adolescent psychiatry and the unique factors that complicate these relationships.

### SSRI Pharmacokinetics in Youths

**FIGURE 1:**
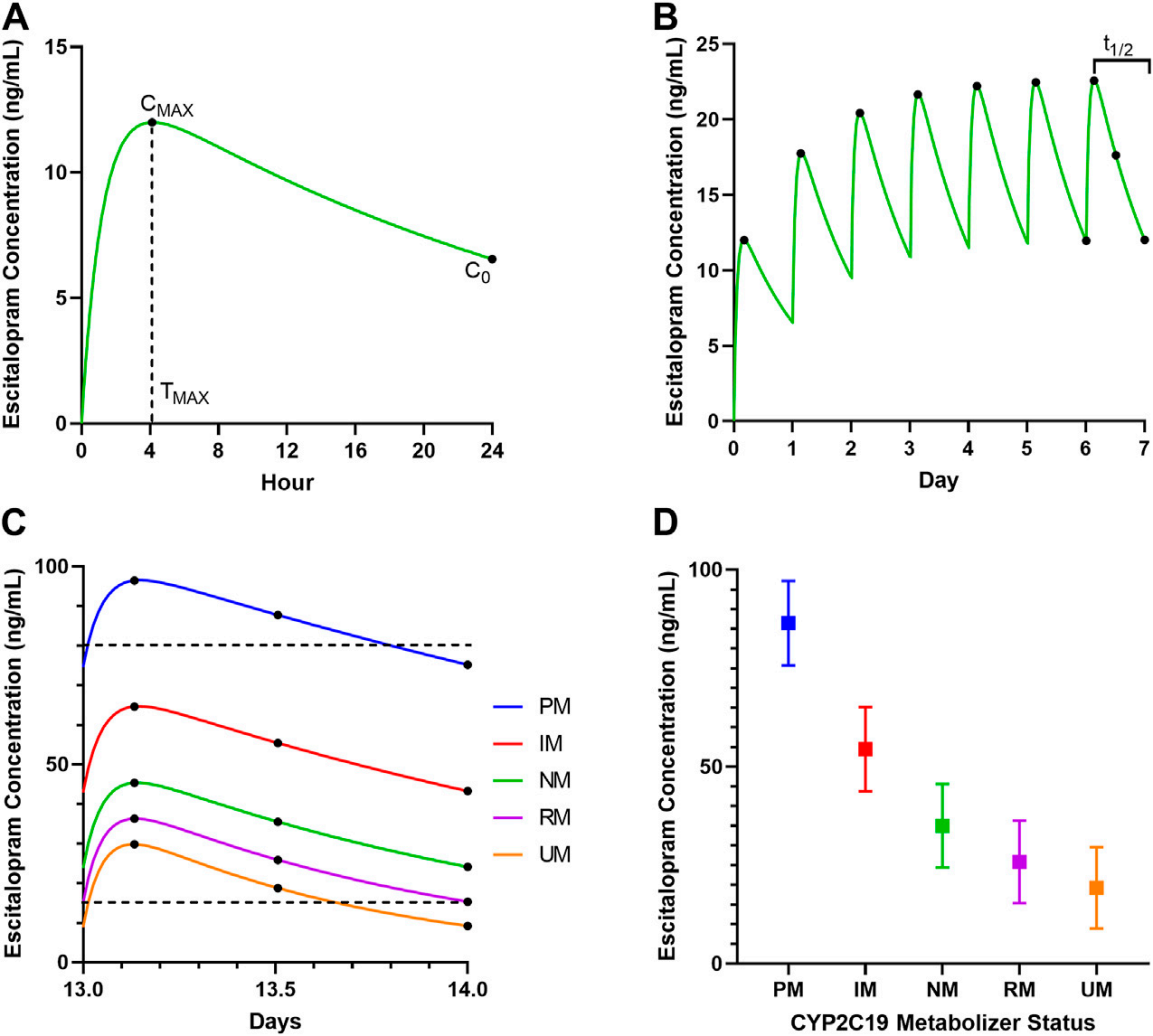
The pharmacokinetics of escitalopram in a 14-year-old adolescent female. **(A)** Predicted concentration-time curve after a single 10 mg dose in a CYP2C19 normal metabolizer. The maximum concentration (*C*_*MAX*_), trough concentration (*C*_*0*_), and time to maximum concentration (*T*_*MAX*_) are shown. **(B)** Concentration-time curve showing seven doses of 10 mg every 24 h in a CYP2C19 normal metabolizer. Dots indicate the *C*_*MAX*_ after each dose, the *C*_*0*_ prior to the seventh dose and the concentrations at 12 and 24 h after the seventh dose. The half-life (t_1/2_) is shown by the bracket above the curve for the seventh dose. **(C)** Escitalopram concentrations after the 14th dose of 20 mg/day are shown for a CYP2C19 poor metabolizer (blue, [PM]), intermediate metabolizer (red, [IM]), normal metabolizer (green, [NM]), rapid metabolizer (purple, [RM]) and ultrarapid metabolizer (orange, [UM]). Dots indicate the maximum concentration after the dose and the concentrations at 12 and 24 h after the 14th dose. Dotted lines indicate therapeutic window (Hiemke et al., 2018). **(D)** For each metabolizer phenotype, the squares indicate the concentration at 12 h after the 14th dose of 20 mg/day, with the whiskers indicating the maximum concentration (*C*_*MAX*_) and the trough concentration (*C*_*0*_).

SSRI exposure is affected by many individual factors (*e.g.*, age, concomitant medications, and cytochrome P450 (CYP) activity), as well as medication dose, amount, and frequency of doses. CYP activity is influenced by genetic polymorphisms affecting the amount and/or function of the protein, age-related changes in the maturation of the enzyme and altered enzyme activity due to specific diseases, as well as inflammation. Understanding the impact of these factors on SSRI pharmacokinetics warrants additional discussion. Consider an adolescent girl with generalized anxiety disorder who is treated with the escitalopram (Figure 1). Following an initial 10 mg dose of escitalopram, her maximal escitalopram concentration (*C*_*MAX*_) is 9.7 ng/ml, the time to the maximal concentration (*T*_*MAX*_) is 4.1 h, and the trough concentration (*C*_*0*_) prior to the next dose is 6.6 ng/ml (Figure 1A). The area under the curve (AUC) is calculated by summing the area under the concentration-time curve between doses or over a certain time frame (*e.g.*, AUC_24_) or until infinity (AUC_∞_). The AUC is dependent on the dose administered and the clearance, and AUC can be calculated by dividing the dose by the clearance. The t_½_ is the time required for a patient to eliminate half the concentration of the drug in the blood. The population average for the t_½_ of most SSRIs is long (*e.g.*, 24 h for escitalopram), so several days are required to reach steady state, and the concentration decreases by half between daily doses.

The patient’s “steady state” occurs when the peaks and troughs are consistent across days because the amount of the medication being added each day is equal to the amount being eliminated from the body each day. Importantly, despite the common misconception, steady state does not indicate that the concentration is consistent between doses. As such, for a medication with a t_½_ of 24 h, the concentration still fluctuates by two-fold each day. Along these lines, some clinicians have argued that t_½_ can be used to determine dosing interval; however, given the variation within a t_½_ (Figure 1), dosing intervals that are less than the t_½_ may be required to maintain consistent exposure above the therapeutic threshold for some SSRIs in youth (Strawn et al., 2019). Steady state is usually achieved after about 4–5 t_½_s of the drug (Figure 1B).

**FIGURE 2:**
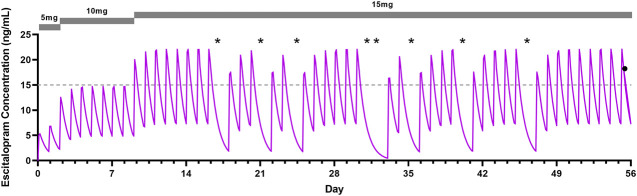
Modeled escitalopram concentration-time profile in a 16-year-old adolescent female CYP2C19 rapid metabolizer with generalized anxiety disorder. Escitalopram dosage is shown in the gray bar (top) and the impact of partial adherence can be seen in the significant decreases in concentration that occurred intermittently beginning in the third week of treatment. The asterisks represent missed doses. The gray dotted-line represents the lower therapeutic threshold for escitalopram (Hiemke et al., 2018). Asterisks represent missed doses, and the black dot reflects the escitalopram determination at the completion of the study.

For some SSRIs in youths, CYP activity—which varies across development (Koukouritaki et al., 2004)—substantially impacts exposure (AUC), *C*_*MAX*_, and t_½._ The impact of CYP2C19 activity on exposure (AUC), *C*_*MAX*_, and t_½_ are shown in Figure 1C. At steady state, after a 20 mg daily dose, CYP2C19 poor metabolizers are likely above the 80 ng/ml toxicity threshold, while ultrarapid metabolizers are likely to be under the 15 ng/ml therapeutic threshold (Hiemke et al., 2018). CYP2C19 activity is also affected by the variability in its expression during growth (e.g. between 5 months and 10 years of age, 21-fold variability is seen) (Kodidela et al., 2017). Moreover, certain disease conditions, such as inflammation, have an impact on CYP2C19 and other CYPs in children above 12 years of age, but not in children below 12 years of age (Koukouritaki et al., 2004). Such age-related differences in enzyme function shall be taken into consideration along with other factors while dosing titrations are being performed. In clinical trials, *C*_*0*_ is often determined prior to a dose, but in clinical practice, patients are often seen between 12 and 24 h after the last dose, and this timing affects SSRI concentrations (Figure 1D). Similarly, adherence has a significant effect on SSRI concentrations (Figure 2). Importantly, failing to account for time since the last dose, the number of previous doses, and adherence introduces substantial variability that obscures the relationship with genotype, metabolizer activity, and response. Yet, many pharmacokinetic models of SSRIs in adults (Shelton et al., 2020) and in youths do not account for many (or all) pertinent covariates (Findling et al., 2006b, 2017; Reinblatt et al., 2009). Failing to account for enzyme ontogeny, allometric scaling or inclusion of the appropriate parameters into these pharmacokinetic models could over or underestimate exposure, which could obscure the relationship between response and exposure or between tolerability and exposure. Inclusion of these covariates in models could help further describe differences in SSRI pharmacokinetics (Figure 1) (Cheung et al., 2019). Such interactions of pharmacogenetics and ontogeny of the enzymes, together with auto- or drug-based enzyme inhibition/induction, must be considered in future investigations to develop precision dosing algorithms.

### TDM and SSRI Pharmacokinetics/Pharmacogenetics in Youths

Relationships between pharmacokinetically-relevant genes (e.g., *CYP2D6* and *CYP2C19*) and SSRI exposure have been established over the past 2 decades. Recently, a meta-analysis of 94 unique studies, revealed significant relationships between CYP2D6 and CYP2C19 metabolizer status and escitalopram, fluvoxamine, fluoxetine, paroxetine and sertraline exposure and reciprocal apparent total drug clearance (Milosavljević et al., 2021). In this meta-analysis, the strongest evidence was for escitalopram and sertraline (Milosavljević et al., 2021). However, only recently has the relationship between SSRI exposure and metabolizer phenotype been explored in pediatric patients, despite preliminary evidence that SSRI exposure may relate to response and tolerability in adolescents with anxiety (Birmaher et al., 2003; Reinblatt et al., 2009; Strawn et al., 2020c) and depressive disorders (Sakolsky et al., 2011).

In a modeling-based simulation of CYP2C19 phenotypes in adolescents, CYP2C19 metabolizer phenotype was associated with differences in escitalopram and sertraline *C*_*MAX*_ and *AUC*_0*-*24_. *C*_MAX_ and *AUC*_0*-*24_ were higher in slower metabolizers (*i.e.*, poor and intermediate metabolizers) and lower in patients with increased CYP2C19 activity, although the magnitude of these differences was more pronounced for escitalopram than for sertraline (Strawn et al., 2019). Additionally, these models may have implications for dosing. For escitalopram, poor metabolizers may require 10 mg/day and ultrarapid metabolizers may require 30 mg/day to achieve an exposure that is equivalent to 20 mg/day in a normal metabolizer. For sertraline, to achieve *AUC*_0-24_ and *C*_*MAX*_ similar to normal metabolizers receiving 150 mg/day, poor metabolizers require 100 mg/day, whereas a dose of 200 mg/day was required in rapid and ultrarapid metabolizers. This raises the possibility that a target concentration could better inform dosing compared to a target dose (Hiemke et al., 2018). These models lend additional support to previously proposed dosing regimens (Findling et al., 2006a). For example, in younger patients and at lower doses, sertraline has a shorter t_*½*,_ raising the possibility that “twice-daily dosing might be reasonable for youths” (Findling et al., 2006a).

Recently, a prospective trial of adolescents with generalized anxiety disorder demonstrated that patients with faster CYP2C19 metabolism (*i.e.*, rapid and ultrarapid metabolizers) had lower escitalopram *AUC*_*0-24*_ (*p* < 0.05) and lower *C*_*MAX*_. Additionally, two studies have examined CYP2C19 phenotype and sertraline and escitalopram concentrations in large pediatric cohorts. In sertraline-treated youths aged 6–17 years (*N* = 107, mean age: 14.5 ± 2.1 years), our group examined sertraline and desmethylsertraline concentrations. Sertraline dose to concentration ratios were decreased in youths with faster CYP2C19 metabolism relative to those with slower metabolism (*p* = 0.002). Fitting of individual patient data to pharmaokinetic models revealed associations between CYP2C19 phenotype and *AUC* and *C*_*MAX*_ (Poweleit et al., 2021). Also, in escitalopram-treated youths (*N* = 104, mean age: 15 ± 1.8 years) escitalopram concentration to dose ratios were decreased in patients with faster CYP2C19 metabolism relative to those with slower metabolism (*p* < 0.001). Also in this sample, escitalopram *AUC*_*0-24*_ significantly decreased with increased CYP2C19 metabolism and *C*_*MAX*_ was higher in slower metabolizers, relative to faster metabolizers (Vaughn et al., 2021b).

One study of single-dose paroxetine pharmacokinetics in youths with depressive disorders (*N* = 30) found “tremendous interindividual variability in paroxetine disposition,” but noted clearance and excretion of paroxetine metabolites correlated with CYP2D6 activity (Findling et al., 1999). Similar findings were reported in a larger multiple-dose study of paroxetine in children and adolescents (*N* = 62, 27 children, 35 adolescents). In this sample, oral clearance was “highly dependent” on CYP2D6 activity, although no association was observed between CYP2D6 phenotype or exposure and adverse events (Findling et al., 2006b). However, the relationship between CYP2D6 activity and exposure in paroxetine- and fluoxetine-treated youths is complicated by phenoconversion (Shah and Smith, 2015). As such, treatment with a strong CYP2D6 inhibitor such as paroxetine or fluoxetine reduces CYP2D6 activity to levels seen in poor metabolizers. The product insert for aripiprazole recommends the same 50% dose reduction for patients that are known CYP2D6 poor metabolizers and those that are taking strong inhibitors of CYP2D6 (CDER FDA, 2014). These patients could possibly benefit from TDM.

### TDM and Drug-Drug Interactions and SSRIs in Youths

Several studies have used modeling-based approaches and *in vivo* data to examine the impact of drug-drug interactions on SSRIs in youths. The nature of this Perspective precludes an extensive review of these studies, including those with cancer patients, transplant patients and critically ill children and adolescents. As such, we will focus on the interaction between two common drug-drug interactions. These were selected given the frequency of their concurrent use with SSRIs in youths and given the increasing use of cannabis (including tetrahydrocannabinol THC) and cannabidiol (CBD) in adolescents.

Both CBD and THC are moderate to strong inhibitors of CYP enzymes (Bansal et al., 2020; Zendulka et al., 2016) and can interact with SSRIs and increase SSRI plasma concentrations. In a small study of es/citalopram-treated adolescents/young adults, aged 17–24 years, CBD significantly increased citalopram plasma concentrations (Anderson et al., 2021). In pharmacokinetic models of adolescents treated with sertraline or escitalopram, CBD and/or THC increase sertraline and es/citalopram *C*_*MAX*_ and *AUC*_*0-24*_ in adolescents (Vaughn et al., 2021a). Additionally, examination of the Food and Drug Administration Adverse Event Reporting System database revealed co-administration of CBD and CYP2C19-metabolized SSRIs increased the risk of some SSRI-related side effects (*e.g.*, diarrhea, dizziness, and fatigue), which may relate to SSRI concentrations (Vaughn et al., 2021a).

Concomitant medications when administered with SSRIs may predispose patients to variation in SSRI plasma concentrations (El Rouby et al., 2018). In adolescents taking some oral contraceptives, steady state plasma citalopram concentrations were significantly affected (Carlsson et al., 2001). This was further confirmed in women taking oral contraceptives and escitalopram in whom metabolite to parent ratios were lower compared to levels in escitalopram-treated women not taking oral contraceptives (Reis et al., 2007). Another study found co-administration of proton-pump inhibitors and SSRIs increased both escitalopram and es/omeprazole plasma concentrations (Gjestad et al., 2015). Given the potential for drug-drug-gene interactions between proton-pump inhibitors, some oral contraceptives, and SSRIs, TDM could help optimize dosing while mitigating the risk of adverse events and reduced response in children and adolescents.

### TDM as a Tool to Assess SSRI Adherence in Youths

TDM has long been used to establish adherence in SSRI-treated adults (Reis et al., 2004, 2010). In fact, in one 6-months sertraline trial using repeated sampling, desmethylsertraline/sertraline ratios were used to identify non-adherence or partial adherence in approximately 10% of the sample (Reis et al., 2004). One pediatric clinical trial has examined concentration-to-dose ratios in youths. In this trial, the Treatment of SSRI-Resistant Depression in Adolescents (TORDIA) study (Brent et al., 2008), the investigators defined a two-fold or greater variation in the dose-adjusted concentrations of the antidepressant medication and metabolite as “non-adherence.” Importantly, in this sample, there was a low concordance between clinician pill counts and concentration-dose ratios, and non-adherence was present in just over half of the participants (Woldu et al., 2011). It is difficult to understate the importance of non-adherence in pediatric patients with anxiety and depressive disorders, as well as other chronic health conditions, especially since average non-adherence across most chronic diseases in youths is near 50% (Walders et al., 2005; Modi et al., 2011).

While TDM has been underutilized in individual patients, it represents a useful tool to understand variation in SSRI exposure and non-response. As an example, the patient described in Figure 2 was a participant in a clinical trial that included measurement of the plasma escitalopram concentrations at the end of treatment. At the 5, 10, and 15 mg daily doses, her *C*_*0*_ was consistently below the therapeutic threshold of 15 ng/ml (based on adult TDM guidelines) (Hiemke et al., 2018) because she was a CYP2C19 rapid metabolizer and had inconsistent adherence at the 15 mg/day dose. However, her phlebotomy was performed after the *C*_*MAX*_, and was above the lower therapeutic threshold. If her escitalopram concentration had been determined just a few days prior, she would not be at steady state, which would need to be accounted for in the analysis.

### Barriers to TDM for SSRIs

In children and adolescents, TDM is frequently restricted to clinical trials and there are significant barriers to its use clinical practice, including a lack of acceptable ‘therapeutic targets,’ pharmacodynamic confounding of exposure-response relationship, long turnaround times for many assays and lower acceptability of phlebotomy in children. Some of these barriers can be overcome with innovations such as opportunistic sampling and dried blood spot analysis that uses only a finger prick of blood for a liquid chromatography mass spectrometry drug concentration measurement (Frey et al., 2020). However, other challenges will require substantial effort and additional research. In addressing these challenges, we may better understand and measure variation in SSRI metabolism, exposure on response and tolerability and ultimately realize dose personalization.

#### Limited Evidence of SSRI Concentration-Effect Relationships

The most significant challenge to TDM arises in the clinic where clinicians frequently assert that therapeutic targets for SSRIs are not well established. Indeed, the lack of established pharmacokinetic-pharmacodynamic relationships for SSRIs in pediatric patients encumbers the routine clinical use of TDM in the clinic. However, this view of TDM and therapeutic reference ranges may be somewhat short-sighted. For many clinicians, the therapeutic reference range specifies a population-based, blood concentration below which a “response is relatively unlikely to occur and an upper limit above which tolerability decreases or above which [additional improvement] is relatively unlikely” (Hiemke et al., 2018). Certainly, some patients improve at concentrations below the therapeutic reference range or fail to develop side effects even when concentrations exceed the therapeutic reference range. Thus, it would behoove us to challenge this conceptualization of TDM as a process to evaluate patients with regard to therapeutic reference ranges for a given medication. The utility of TDM for SSRIs in youth may be conceptualized as a continuum of applications—a view consistent with the Consensus Guidelines for Therapeutic Drug Monitoring in Neuropsychopharmacology (Hiemke et al., 2018). Using this approach, the utility of TDM in SSRI-treated youths could be seen as Level 3 (reference ranges are unavailable or based on non-systematic clinical experience) or Level 4 (exposure does not correlate with response or tolerability because of “unique pharmacology of the drug, e.g., irreversible blockade of an enzyme, or dosing can be easily guided by clinical symptoms”) (Hiemke et al., 2018).

#### Pharmacodynamic Confounding of SSRI Concentration-Effect Relationships

**FIGURE 3:**
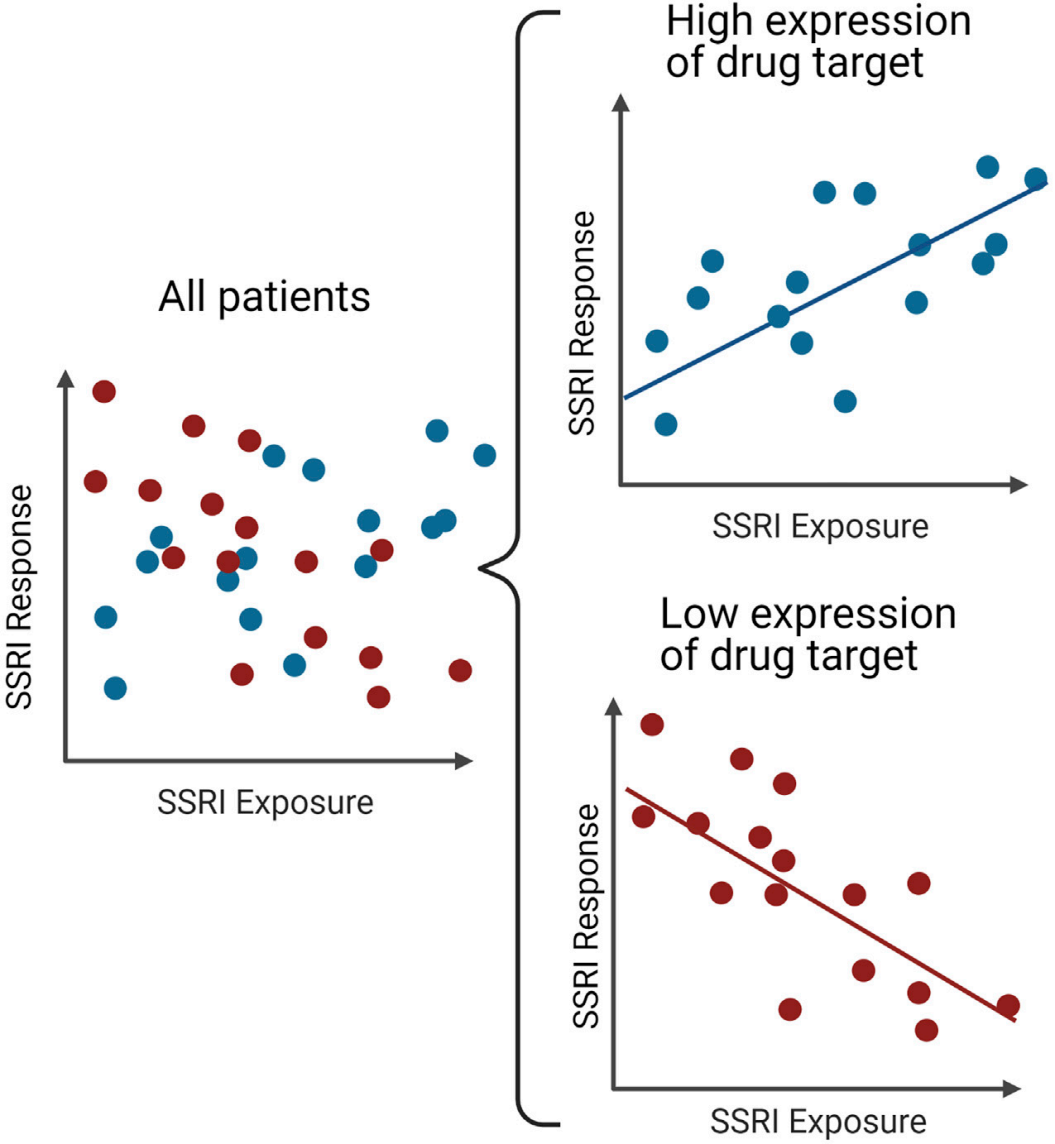
Pharmacodynamic confounding of the SSRI exposure-response relationship. In a combined sample of SSRI treated patients (left), there does not appear to be a relationship between SSRI response and exposure. However, when patients are examined separately, based on a pharmacogenetic variant that impacts pharmacodynamics (e.g., *SLC6A4*), some patients have a positive relationship between response and SSRI exposure whereas other patients—such as those with low expression of the drug target—have a negative correlation between response and SSRI exposure, as per the example in the text of paroxetine and *SLC6A4*.

Another challenge to establishing therapeutic reference range and the ‘lack’ of relationships between exposure and response is pharmacodynamic confounding of the exposure-response relationship (Figure 3). There is variation in expression of the SSRI target, the serotonin transporter (encoded by *SLC6A4*) that has been associated with genetic variants (Zhu et al., 2017). In one study of adults treated with paroxetine (Tomita et al., 2014), there was no apparent exposure-response relationship until the patients were divided into those predicted to have high expression of the drug target (L allele carriers) and those predicted to have low expression (SS genotype). In the high expression group, the expected positive association was seen between exposure and response. In the low expression group, the opposite was seen, likely due to adequate blockage of the transporter at low exposure and off-target effects at higher exposure. It’s difficult to evaluate an exposure-response relationship without accounting for both pharmacokinetic and pharmacodynamic variability (Hertz et al., 2021).

#### Developmental and Disease State Influences on SSRI Concentration-Effect Relationships

Therapeutic reference ranges may also vary developmentally and be indication specific (Egberts et al., 2011). Further, therapeutic reference ranges may change based on the phase of the illness, as with other chronic, relapsing-remitting disorders (i.e., higher exposure required during acute exacerbations relative to maintenance phases). How variation in exposure, whether related to intrinsic factors (*e.g.*, metabolism) or extrinsic factors (*e.g.*, adherence), influences multivariate predictive models of response and has received limited attention. Understanding the interaction of family factors, disease state, age, inflammation/acute systemic illness, trauma exposure and co-morbidity is critical to refining and applying TDM-informed predictive models for both efficacy and tolerability.

#### Traditional SSRI Dosing Approaches as Barriers to TDM

Many presume that exposure can be inferred from a “start low and go slow” approach. However, this approach of standardized initial dosing and titration still places poor metabolizers at risk for side effects given a 3-fold higher exposure (Jukić et al., 2018), and may result in a protracted course for some patients as achieving effective exposure in faster metabolizers requires substantially more time. A second challenge involves the clinical assertion that side effects are unrelated to variation in exposure. Yet, from a tolerability standpoint, variation in pharmacokinetic genes—which produces variation in exposure—has been associated with SSRI tolerability and relationships have been established for escitalopram-related activation and weight gain (Aldrich et al., 2019; Strawn et al., 2020c). Further work is needed to assess this paradigm, especially since retrospective evaluation of SSRI tolerability in pediatric patients has contrasted that of adults (Poweleit et al., 2019; Rossow et al., 2020).

### Future Directions

While TDM in SSRI-treated children and adolescents is in its early stages, multiple applications can already be imagined, including evaluating adherence and establishing probabilistic models that identify patients who are at the highest risk of side effects or who require higher doses or alternative dosing regimens (*e.g*., twice vs. once daily). Another opportunity lies in the advent of big data and machine learning to provide predictions that act as a surrogate or complement to traditional pharmacometrics. Machine learning and artificial intelligence can serve as a “computational bridge between big data and pharmacometrics,” with specific applications towards TDM (*e.g.*, pharmacokinetics/pharmacodynamics and dose optimization) (McComb et al., 2021). Development of tools that allow clinicians to input individual patient characteristics to predict their SSRI concentration comparable to current pharmacokinetic modeling could overcome some barriers of TDM for SSRIs (*e.g.,* the need for phlebotomy, long turnaround times for assays). While further work is needed, machine learning applications have the potential to provide generalizable and autonomous TDM predictions for SSRIs in youths.

## Data Availability

The original contributions presented in the study are included in the article, further inquiries can be directed to the corresponding author.
